# Enhanced Optoelectronic Performance of Yellow Light-Emitting Diodes Grown on InGaN/GaN Pre-Well Structure

**DOI:** 10.3390/nano11123231

**Published:** 2021-11-28

**Authors:** Xiaoyu Zhao, Zehong Wan, Liyan Gong, Guoyi Tao, Shengjun Zhou

**Affiliations:** 1 Center for Photonics and Semiconductors, School of Power and Mechanical Engineering, Wuhan University, Wuhan 430072, China; xy.zhao@whu.edu.cn (X.Z.); 2019202080016@whu.edu.cn (L.G.); 2 The Institute of Technological Sciences, Wuhan University, Wuhan 430072, China; wanzh17@whu.edu.cn (Z.W.); tao.gy@whu.edu.cn (G.T.)

**Keywords:** gallium nitride, yellow LED, pre-well structure, optoelectronic device

## Abstract

InGaN-based long-wavelength light-emitting diodes (LEDs) are indispensable components for the next-generation solid-state lighting industry. In this work, we introduce additional InGaN/GaN pre-wells in LED structure and investigate the influence on optoelectronic properties of yellow (~575 nm) LEDs. It is found that yellow LED with pre-wells exhibits a smaller blue shift, and a 2.2-fold increase in light output power and stronger photoluminescence (PL) intensity compared to yellow LED without pre-wells. The underlying mechanism is revealed by using Raman spectra, temperature-dependent PL, and X-ray diffraction. Benefiting from the pre-well structure, in-plane compressive stress is reduced, which effectively suppresses the quantum confined stark effect. Furthermore, the increased quantum efficiency is also related to deeper localized states with reduced non-radiative centers forming in multiple quantum wells grown on pre-wells. Our work demonstrates a comprehensive understanding of a pre-well structure for obtaining efficient LEDs towards long wavelengths.

## 1. Introduction

III-nitride emitters have attracted a lot of attention due to their advantages of energy savings, high brightness, and long lifetime. With the wide and tunable band gap, InGaN-based light emitting diodes (LEDs) find widespread applications in the solid-state lighting and full-color display [1,2,3,4,5,6,7]. In recent years, the increasing need of flexible lighting devices motivates the manufacturing techniques development for deformable micro-LEDs [8] and the progress of flexible micro-LEDs applications in the optogenetic biomedical field [9]. Though blue LEDs achieving a high external quantum efficiency [10], the emission efficiency is still limited in the long-wavelength region, which is commonly known as “green-yellow gap” phenomenon [2]. One of the main reasons arises from the strong piezoelectric field along the (0001) direction induced by the large lattice mismatch between sapphire substrate and epilayers. This built-in piezoelectric field gives rise to quantum-confined stark effect (QCSE) [11], which separates electron-hole wavefunctions and further degrades the radiative recombination efficiency. When the In content increases, QCSE becomes more severe, hindering the pursuit for efficient LEDs with long wavelengths.

Numerous efforts have been dedicated to improving the InGaN-based LED performance in the long-wavelength region, such as introducing semipolar/nonpolar substrate [12], inserting strain engineering layers [13,14], and adopting bandgap engineering quantum wells [15,16]. Among them, the strain engineering strategy, which utilizes low In content layers prior to the growth of active region, is an effective and low-cost method, also known as the “pre-layer structure”. Huang et al. found that a green LED structure with pre-strained growth showed enhanced efficiency and reduced spectral shifts, which could be attributed to decreased defect density and the reduced QCSE [17]. Niu et al. reported that insertion of an InGaN layer increased photoluminescence (PL) intensity more than twice as much, and improved crystal quality [18]. The inclusion of InGaN/GaN superlattice pre-layers could facilitate more In incorporation and extend emission wavelength, as Hu et al. demonstrated [19]. The pre-layer structure could act as the buffer layer to relax in-plane strain in multiple quantum wells (MQWs). Until now, while various pre-layers have been extensively discussed in blue and green region [14,17,18,19,20,21], their epitaxial structures differ much from corresponding MQWs. Besides, the influence of pre-layer structure on yellow LEDs performance remains insufficient [13,22], especially in terms of some related physic issues, including the effect of carrier localization.

In this work, we adopt an InGaN/GaN pre-well structure between superlattices and active region, and study its influence on optoelectronic characteristics of yellow (~575 nm) LEDs. At the same injection current, LED with pre-wells achieves a smaller blue shift and a higher light output power (LOP) than those of LED without pre-wells. Raman spectra show that relaxation of compressive strain is achieved inside MQWs grown on InGaN/GaN pre-wells, which alleviates the negative influence of QCSE. Additionally, through time-dependent photoluminescence (TDPL) and X-ray diffraction (XRD) analysis, localized states are found to be more pronounced in MQWs grown on pre-well structure, owing to the decreased dislocation density. Due to these advantages, yellow LED with pre-wells exhibits improved optical and electrical properties, enabling the development for efficient III-nitride emitters.

## 2. Experiments

**Figure 1:**
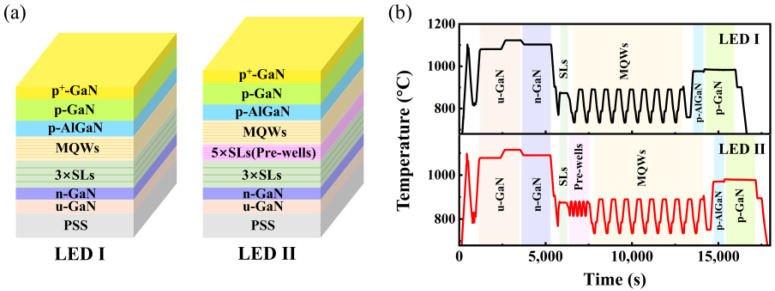
(**a**) Schematic diagram of the epitaxial structure of LED I and LED II. (**b**) Temperature profile during the growth process of LED I and LED II.

The LED samples were grown on the patterned sapphire substrate (PSS) via metal organic chemical vapor deposition (MOCVD). The schematic diagram of two types of yellow LED structure is presented in Figure 1a. The reference LED (LED I) started from a 3.0-µm-thick undoped GaN layer. Subsequently, a 2.0-µm-thick Si-doped n-GaN layer was deposited, followed by three pairs of In_0.01_Ga_0.99_N (7 nm)/GaN (50 nm) superlattices (SLs). The active region consists of nine pairs of In_0.1_Ga_0.9_N (0.6 nm)/In_0.35_Ga_0.65_N (1.3 nm)/In_0.1_Ga_0.9_N (0.6 nm)/GaN (13 nm). Then, a 25-nm-thick low-temperature p-GaN layer was capped on MQWs. Then, a 20-nm-thick AlGaN electron-blocking layer and a 50-nm-thick Mg-doped p-GaN layer were grown, ending with a 5-nm-thick heavily Mg-doped p^+^-GaN. For the optimized LED (LED II), prior to the growth of MQWs, five pairs of In_0.05_Ga_0.95_N (2 nm)/GaN (10 nm) SLs, named as pre-wells, were deposited on three pairs of SLs. Notably, different from aforementioned pre-layer structures, the thicknesses of pre-wells in this work was intentionally designed to be similar to MQWs, with an aim to further reduce the lattice mismatch between underlayers and MQWs. Figure 1b shows the temperature profile during the growth of two LED structures, in which the only difference is the additional growth of pre-wells. A detailed description of fabrication of LED chips has been provided in our previous work [23]. The LED chips were fabricated with areas of 239 μm × 356 μm.

To characterize the epitaxial structure of LED samples, we carried out the cross-sectional transmission electron microscopy (TEM), atom probe tomography (APT), Raman spectroscopy, and X-ray diffraction (XRD) measurements. The light output power–current–voltage (L-I-V) characteristics of the yellow LEDs were measured by an integrating sphere together with a semiconductor parameter analyzer at room temperature. TDPL measurements were performed using a 325-nm He–Cd laser with an excitation power of 5 mW.

## 3. Results and Discussion

**Figure 2:**
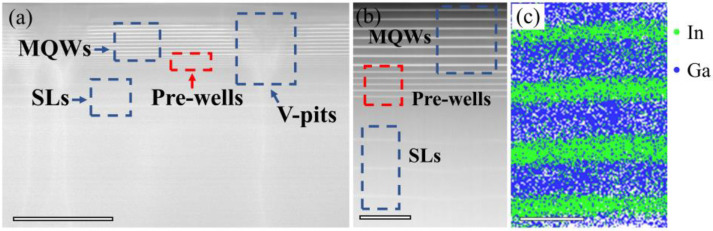
(**a**,**b**) Cross-sectional TEM images of epitaxial structure for LED II. (**c**) Reconstructed atom map of MQWs in LED II. Scale bars, 500 nm (**a**), 100 nm (**b**), 18 nm (**c**).

Figure 2a,b show the cross-sectional bright-field TEM images of epilayer structure with pre-wells. Each layer in LED II exhibits uniform thickness and clear interface, albeit the difference in brightness contrasts among SLs, pre-wells, and MQWs, as seen in Figure 2a,b. Besides, from Figure 2a, we observe that V-shaped pits form in SLs and extend to MQWs. Our previous work found that optimizing the density and size of V-pits could contribute to the increased emission efficiency by suppressing non-radiative recombination [23]. Figure 2c shows the reconstructed atom map of MQWs of LED II via APT measurement, showing the distribution of In and Ga elements.

**Figure 3:**
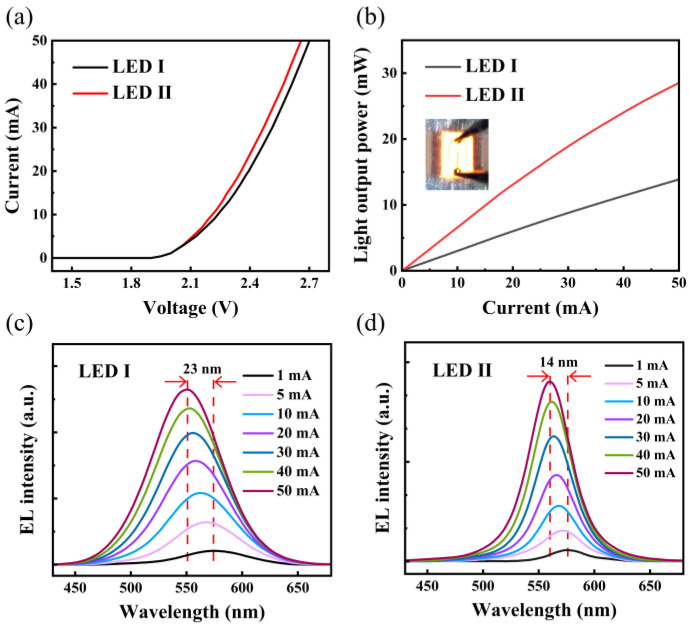
(**a**) I-V characteristics for sample I and sample II. (**b**) L-I characteristic for sample I and sample II. Normalized EL spectra for (**c**) LED I and (**d**) LED II at room temperature with increasing forward currents, respectively.

Figure 3a,b show the current–voltage (I-V) and light output power–current (L-I) characteristics of the yellow LEDs, respectively. In Figure 3a, the forward voltages for LED I and LED II are 2.39 V and 2.36 V at 20 mA, respectively. This indicates that the pre-well structure has a negligible impact on the electrical properties. Note that LED II has an enhanced LOP in compared with LED I. At 20 mA, the LOP for LED I is 5.9 mW, while that for LED II is 13 mW. Room-temperature electroluminescence (EL) measurements were performed and shown in Figure 3c,d. As the injection current increases, the peak wavelengths for two samples are observed in the blue shift, which arises from the charge screening of polarization field and band-filling effect [24]. It could be observed that LED II shows a much smaller blue shift (~14 nm) than LED I (~23 nm) when the injection current increases from 1 mA to 50 mA. For most InGaN MQWs grown on c-plane sapphire, they undergo QCSE which causes strong band tilting and wavelength shifting. As the carriers are injected, the built-in electric field are screened, leading to a decreased wavelength shift. Thus, we believe that, with the inclusion of a pre-well structure, a negative impact of QCSE is alleviated, and the LED with pre-wells achieves a smaller blue shift. Besides, from the EL spectra, there is no additional peak induced by the emission in In_0.05_Ga_0.95_N/GaN pre-wells, implying that a recombination in MQWs dominates the recombination process in our designed structure.

**Figure 4:**
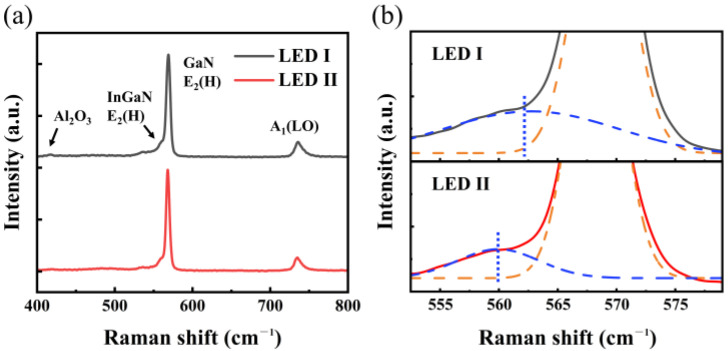
(**a**) Raman spectra for LED I and LED II. (**b**) Enlarged view of the E_2_ (H) mode peak. Blue and orange dashed lines show the separated InGaN and GaN E_2_ (H) modes, respectively.

In order to reveal the underlying mechanism of improved performance, we carried out the confocal micro-Raman measurement, as shown in Figure 4. As the E_2_ (H) mode is sensitive to the strain [25], it is widely utilized to assess the residual stress in epitaxial structure. However, considering the pre-well structure located between the thick GaN layer and MQWs, the InGaN E_2_ (H) mode is suitable for evaluating the residual stress in MQWs. In the Raman spectra, there are two peaks positioned at ~569 cm^−1^ and ~560 cm^−1^, which originate from the GaN E_2_ (H) and InGaN E_2_ (H) mode, respectively [26]. In particular, the InGaN E_2_ (H) mode decreases from 562.7 cm^−1^ (LED I) to 559.9 cm^−1^ (LED II). It indicates that the adoption of pre-wells contributes to stress relaxation [26], which could be attributed to the similar structure between pre-wells and MQWs. It is well known that QCSE separates the electron and the hole wavefunctions towards the opposite direction and reduces the electron-hole wavefunction overlap, thereby deteriorating the radiative recombination rate [15]. By adopting pre-well, the negative impact of stress-induced QCSE is suppressed and, more so, the quantum efficiency is enhanced. As a result, LED II achieves improved LOP as well as the smaller blue shift (Figure 3).

**Figure 5:**
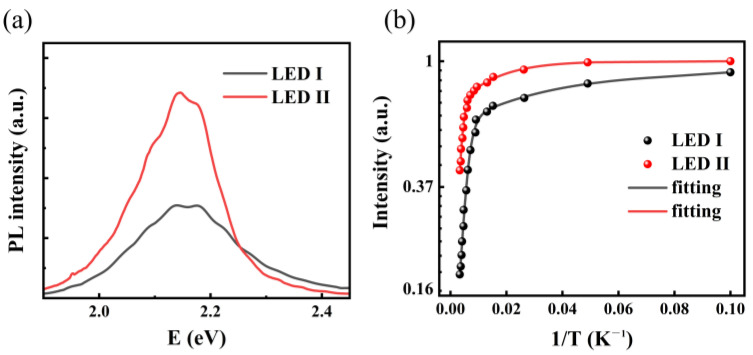
(**a**) PL spectra at room temperature for LED I and LED II. (**b**) Normalized integrated PL intensity as a function of 1/T for LED I and LED II, along with the Arrhenius fitting plots.

The room-temperature PL spectra are shown in Figure 5a. LED II possesses a higher PL peak intensity than that of LED I, which means enhanced radiative recombination by introducing pre-well structure. Figure 5b shows the TDPL spectra. The PL intensity of LEDs dramatically decreases with increasing temperature. Such thermal quenching of PL intensity results from the pho-non-assisted non-radiative recombination and the integrated PL intensity versus temperature can be fitted by using the Arrhenius formula [27]:

(1) $$I\left(T\right)=I_{0}/\left[1+\sum_{i}C_{i}\exp\left(-E_{\mathrm{Ai}}/k_{B}T\right)\right]$$

where I (T) represents the normalized integrated PL intensity, C_i_ represents the constants, and E_Ai_ represents the activation energies correlated with nonradiative recombination process. In particular, E_A1_ and E_A2_ can be attributed to (1) localized exciton binding energy, and (2) the potential barrier between the localized potential minima and nonradiative centers located within MQWs, respectively [28]. Through the curve fitting, the yielding activation energies are 3.04 meV (E_1_) and 53.68 meV (E_2_) for LED I, while those for LED II are 6.89 meV (E_1_) and 85.64 meV (E_2_), respectively. Since the obtained activation energies are much lower than the band offsets between wells and barriers, thermal quenching of PL intensity results from the delocalization of excitons [29]. The activation energies often reflect the localization energy of localized states [30], in which carriers could be trapped and have less possibility to meet other non-radiative recombination centers. Accordingly, we suppose pronounced localized states existing in the pre-well structure, which provides strong confinement for carriers and preventing them from being captured by non-radiative recombination centers.

**Figure 6:**
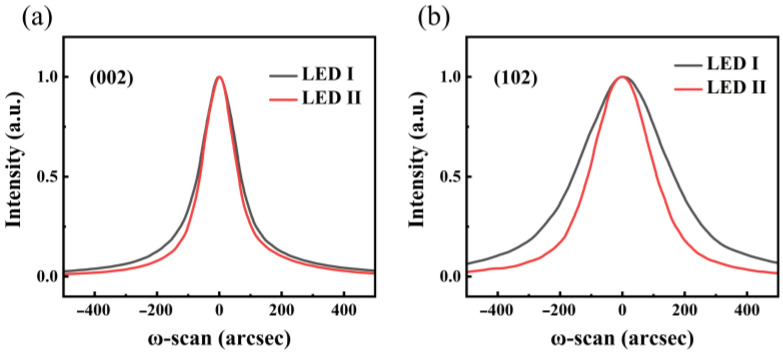
XRD rocking curves of (**a**) (002) and (**b**) (102) planes for LED I and LED II.

To elucidate our finding, XRD measurements were performed to assess the non-radiative centers density existing in the two samples. It is acknowledged that the full width at half maximum (FWHM) of XRD ω-rocking curves for different planes could reflect the specific threading dislocation, including screw dislocation and the edge dislocation in the epitaxial structure. Figure 6a,b shows the XRD ω-rocking curves for (002) and (102) planes of two samples, respectively. We find that, in LED II, FWHMs of ω-rocking curves for both (002) and (102) planes are 147.50 and 243.18 arcsec, respectively, while they are 176.77 and 363.93 arcsec, respectively, in LED I. Threading dislocation density (TDD) in two samples is roughly estimated through the formula [31]:

(2) $$N=\frac{\beta^{2}}{4\times {\left|b\right|}^{2}}$$

where N represents the TDD, β represents the FWHM of the rocking curve, and b represents the Burgers vector of the corresponding dislocations. The total TDDs in LED I and LED II are estimated to be 8.34 × 10^8^ and 3.89 × 10^8^ cm^−2^, respectively. This implies that threading dislocations, acting as non-radiative centers, are reduced by adopting pre-wells. Carrier localization has been reported to occur in the direct vicinity of the dislocation through the formation of In–N chains and atomic condensates [32]. In particular, the clustered dislocations tend to screen the effects of carrier localization, and thus the LED I with more TDD, displays the weakened carrier localization compared to LED II, leading to the inferior quantum efficiency.

## 4. Conclusions

In summary, we make comprehensive analysis of InGaN-based yellow (~575 nm) LED performance by adopting the InGaN/GaN pre-well structure. LED with pre-wells exhibits better optoelectronic properties, especially an increase in LOP of 13 mW, which is 2.2 times higher than that for LED without pre-wells at 20 mA. Experiment results reveal that this structure plays a vital role for stress relaxation. The performance enhancement is attributed to alleviated detrimental influence of QCSE and strengthened carrier localization in MQWs grown on pre-wells. Our work gains insight into the influence of pre-well structure on InGaN-based long-wavelength LEDs, by presenting their advantages for the future solid-state lighting.

## Data Availability

Data are contained within the article.
